# Novel Truncating Variants in *PODXL* Represent a New Entity to Be Explored Among Podocytopathies

**DOI:** 10.3390/genes16040464

**Published:** 2025-04-18

**Authors:** José María García-Aznar, María Lara Besada-Cerecedo, Cristina Castro-Alonso, Milagros Sierra Carpio, Miquel Blasco, Borja Quiroga, Michal Červienka, Ricardo Mouzo, Roser Torra, Alberto Ortiz, Patricia de Sequera

**Affiliations:** 1 Clinical Area of Genetic Diagnostic in Nephrology, Healthincode, 15008 A Coruña, Spain; 2 Department of Nephrology, Doctor Peset University Hospital, Fundación para el Fomento de la Investigación Sanitaria y Biomédica de la Comunitat Valenciana (FISABIO), 46020 Valencia, Spain; 3 Nephrology Department, Hospital Universitario San Pedro, 26006 Logroño, Spain; 4 Nephrology and Kidney Transplant Department, National Reference Center for Complex Glomerular Diseases (CSUR), Hospital Clínic, Barcelona University, 08036 Barcelona, Spain; 5 Fundació de Recerca Clínic Barcelona-Institut d’Investigacions Biomèdiques August Pi i Sunyer (FRCB-IDIBAPS), 08036 Barcelona, Spain; 6 Nephrology Department, Hospital Universitario de la Princesa, 28006 Madrid, Spain; 7 Nephrology Department, Hospital El Bierzo, 24404 Ponferrada, Spain; 8 Nephrology Department, Fundació Puigvert, 08025 Barcelona, Spain; 9 Nephrology and Hypertension Department, IIS-Fundación Jiménez Díaz UAM, 28040 Madrid, Spain; 10 Medicine Department, Facultad de Medicina, Universidad Autónoma de Madrid, 28029 Madrid, Spain; 11 Nephrology Department, Hospital Universitario Infanta Leonor, 28031 Madrid, Spain; 12 Medicine Department, Facultad de Medicina, Universidad Complutense de Madrid, 28040 Madrid, Spain

**Keywords:** CKD 1, PODXL 2, podocytopathy 3

## Abstract

Background/Objectives: Podocalyxin is a sialoprotein mainly expressed in the kidney cortex and lung tissue, which has been described as a component of the podocyte glycocalyx. This protein promotes the reorganization of the podocyte cytoskeleton, as well as the morphogenesis and differentiation of nascent podocytes, actively participating in glomerular filtration. Previous research has suggested that *PODXL* haploinsufficiency leads to podocytopathy with development of focal segmental glomerulosclerosis, a disorder that has been demonstrated in Podxl-deficient animal models and proposed as a primary cause in human families affected by this condition. However, only a few families have been reported, which limits the understanding about the spectrum of phenotype and prognosis of the disease. Methods: We performed high-throughput sequencing in a cohort of young adults with CKD, describing the clinical scenario of those who harbored truncating variants in the *PODXL* gene and testing the families for detected variants. Results: The *PODXL* gene exhibited a slight deviation in loss intolerance probability and moderate deviation in the observed/expected ratio of variation, which is typically observed in dominant genes with age-dependent incomplete penetrance or variable expression. We reported four novel truncating variants in the *PODXL* gene, along with a collection of previously published monoallelic truncating variants. Conclusions: These findings further support evidence about genetic defects in the *PODXL* gene associated with a new molecular entity of podocytopathy with adult onset. Additionally, the nucleotide sequence of *PODXL* contains particularities that require careful analysis to interpret the effect of the variants detected in this gene.

## 1. Introduction

The existence of podocalyxin was first described in 1987 when a single polyanionic sialoprotein of approximately 140 kDa was isolated from the surface of the glomerular epithelium. It was observed to be the main constituent of the podocyte glycocalyx, and it is characterized by the presence of distal groups of *Gal-Ga1NAc* susceptible to being O-glycosylated [1]. Cloning and human sequencing studies of the *PODXL* gene revealed that this gene contains a signal peptide within the first 22 amino acids, with an upstream GC-rich region (amino acids 1–22); an extensive extracellular domain (amino acids 23–431) harboring five potential *N*-glycosylation sites and multiple potential *O-*glycosylation sites; a hydrophobic transmembrane domain (amino acids 462–482); and a *COOH*-terminal region with cytoplasmic localization (amino acids 463–558), which includes two potential casein kinase II phosphorylation sites [2].

**Figure 1:**
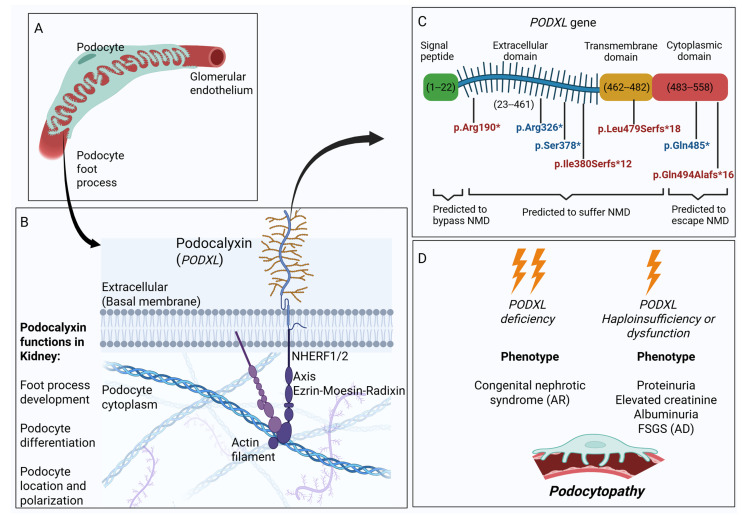
(**A**) Podocytes wrapped around a glomerular capillary. (**B**) Podocalyxin functions in kidney and *PODXL*–protein interaction. The cytoplasmic domain of podocalyxin recruits NHERF-1/2, which interact with the ERM (ezrin–moesin–radixin) axis through the phosphorylation sites located in its *COOH* terminal region, promoting the polymerization of the actin cytoskeleton in foot processes of podocytes. (**C**) *PODXL* gene structure representation by domains whose positions have been extracted from www.nextprot.org. Previously described truncating variants in the *PODXL* gene are represented in blue, whereas the novel truncating variants described in this study are shown in red. The prediction about the escaping/susceptibility to NMD of the truncating variants was established based on the functional studies and location of the premature stop codon across the *PODXL* sequence. (**D**) Related phenotypes produced by complete deficiency or haploinsufficiency/disruption of podocalyxin.

Bulk expression data from Genotype-Tissue Expression (GTEx project) have shown that podocalyxin is mainly present in the renal cortex and in the lungs at maximum TPM (transcripts per million) values of 161.5 and 133.5 TPM, respectively [3]. To understand whether the deficiency of this protein triggers a kidney disease, the most updated version of gnomAD (v4.1.0) indicates that *PODXL* exhibits a slight deviation in loss intolerance probability [pLI = 0.27] and a moderate deviation in the observed/expected ratio of variation [o/e = 0.41 (0.29–0.6)], suggesting partial intolerance to loss-of-function (LoF) variants [4]. Monoallelic truncating variants in the *PODXL* gene have been proposed to cause podocytopathy, as podocalyxin activates the RhoA pathway and induces the actin reorganization of the podocyte cytoskeleton, directly involved in the morphogenesis and differentiation of nascent podocytes. Podocalyxin promotes the transition of tight and adherent junctions and the subsequent formation of foot processes and cleft diaphragms, key ultrastructural elements that enable glomerular filtration [5]. In this regard, at least three disease-causing nonsense variants have been reported (p.Arg326*, Ser378*, and p.Gln485*) in families with autosomal dominant focal segmental glomerulosclerosis [6,7]; moreover, variants p.Met1Ile and p.Trp341* have been found in compound heterozygosis in a patient with congenital nephrotic syndrome, omphalocele and microcoria [6]. Previous murine models of podocalyxin deficiency (*Podxl^−^/^−^*) showed it to be perinatally lethal within one day after birth, causing a complete loss of *PODXL* expression [8]. At examination, these mice showed edema and omphalocele as gross anomalies and anuric renal failure caused by a lack of foot processes and slit diaphragms, which reduced glomerular permeability and impaired filtration capacity [9]. Thus, complete deficiency of PODXL leads to severe congenital renal disease affecting critical processes of podocyte development, while its haploinsufficiency results in end-stage renal disease due to podocyte dysfunction (Figure 1).

Another relevant aspect of podocalyxin is its involvement in non-renal systems. Podxl-deficient mice developed nonspecific vasculitis with respiratory distress caused by decreased pulmonary vascular permeability. The loss of *PODXL* alters endothelial cell adhesion, producing severe lesions in the vascular bed, likely linked to its interaction with the ezrin–moesin–radixin complex through the PODXL *COOH*-terminal domain, which is a known modulator of the endothelial cell barrier [10]. The consequences of the endothelial injury would activate thrombin-dependent machinery and inflammatory processes. It has also been proposed that PODXL is required for early vascular lumen formation in the aorta [11]. Furthermore, PODXL is also expressed in the brain, where its deficiency induces the activity of ABCB1/MDR1, involved in the control of tight junctions and immune responses [12]. Recently, the role of PODXL as a cause of autosomal recessive juvenile Parkinsonism is under discussion [13].

In this study, we report four novel truncating mutations in the *PODXL* gene associated with the development of glomerular disease, which, along with other previously described variants, contribute to a deeper understanding of the mutational landscape of the *PODXL* gene.

## 2. Materials and Methods

### 2.1. Cohort Selection

We systematically sequenced and analyzed variants in the *PODXL* gene in 818 patients that developed end-stage kidney disease (ESKD) before age 45. This cohort of patients were sequenced as part of the GENSEN project, promoted by the Spanish Society of Nephrology (S.E.N.) [14]. The inclusion/exclusion criteria of this study included patients who developed kidney failure at age 45 years or younger, whose physicians considered that the cause of CKD remained undefined, and who had CKD category G5 (GFR < 15 mL/min/1.73 m^2^) or were undergoing either dialysis or transplantation. Some patients with early CKD before age 45 who did not develop CKD-V stage were analyzed in this cohort (36 patients).

We supported statistical results about truncating mutations in the *PODXL* gene with anonymized data from 4408 sequenced individuals belonging to a cohort of patients affected by immunological, neurological, cardiovascular, and renal diseases.

### 2.2. Molecular Analysis

The genetic study was performed using a customized 529-gene HTS target-gene capture library, according to our previous publication [15]. DNA was extracted from peripheral blood or saliva samples using automated genomic deoxyribonucleic acid (DNA) purification (QIAsymphony SP^®^, Qiagen, Hilden, Germany). Samples were prepared using the Agilent SureSelect Library Preparation Kit (Santa Clara, California, USA) for Illumina (San Diego, CA, USA) paired-end multiplexed sequencing according to the manufacturer’s instructions.

Bioinformatics analysis was performed in accordance with the best WES analysis practices. Genetic variants were aligned using the reference genome GRCh37/hg19 and were reported following the Human Genome Variation Society (HGVS) recommendations (www.hgvs.org). All the variants identified in the *PODXL* gene were confirmed by Sanger sequencing.

### 2.3. Review of PODXL Variants

We searched the scientific literature for reported *PODXL* mutations potentially causing renal disease, mainly, but also related to other human pathologies. We employed the following keywords in PubMed: “PODXL”; “variants/mutation”; and “Podocalyxin”. Sixty-three indexed publications were found. From them, we selected four articles in which variants in the *PODXL* gene were associated with renal disease in order to describe the patients reported in the literature with potentially pathogenic variants in *PODXL* gene.

The review was completed by searching the following human mutation databases: ClinVar and Leiden Open Variation Database (LOVD). We restricted this search to truncating pathogenic or likely pathogenic variants (Supplementary Materials).

## 3. Results

### 3.1. Patient Description

– Case 1: A 62-year-old male patient with a medical history of grade I obesity, hypertension, dyslipidemia, and hyperuricemia was referred for nephrology consultation at the age of 40 after proteinuria was detected in the range of 1–2 g/24 h. No previous urine tests were available. He had normal serum albumin and preserved kidney function. An immunological study (ANA, ANCA, antiMBG antibodies, IgG, IgM, IgA, C3, C4, rheumatoid factor, RCP, PLA2R antibody, and serum proteinogram) was carried out and yielded negative results. He was started on olmesartan 40 mg. During subsequent follow-up, the same degree of proteinuria was maintained with slight variations until April 2023, when proteinuria was raised to 5 g/24 h, with normal serum albumin and preserved renal function. Dapagliflozin 10 mg was added to his treatment regimen. Results from optic microscopy and immunofluorescence following kidney biopsy revealed alterations compatible with focal segmental glomerulosclerosis (FSGS), absence of immune deposits, and tubular atrophy/interstitial fibrosis (15–20%). Spironolactone 25 mg was added, and genetic testing was requested. In subsequent follow-up, a progressive decrease in proteinuria up to 1.88 g/24 h was observed, maintaining kidney function and serum albumin within normal ranges. The phenotype of the patient was not observed in any other family members.
– Case 2: A 50-year-old male patient with a history of recurrent tonsillitis since childhood. At age 22, hypercholesterinemia (284 mg/dL) and elevated creatinine (2.1 mg/dL) were detected. Urinalysis showed proteinuria 2.7 gr/day and microhematuria, while immunological study (IgG, IgG, IgA, M, ANA, C3–4, and ICC) was normal/negative. Urine culture and smears were negative. Renal ultrasound imaging revealed decreased kidneys size with increased echogenicity at the cortex level. Intravascular ultrasound displayed a lobulated appearance of the kidneys. He also was evaluated by rheumatology because of a left sacroiliitis diagnosis and a positive HLA B27 test. He experienced progressive deterioration of renal function, starting peritoneal dialysis at age 30. At the age of 32, he was referred for cadaveric renal transplantation. There was no evidence of renal involvement in his parents and other family members.
– Case 3: A 55-year-old male patient with unaffiliated chronic kidney disease. At the age of 26, high blood pressure was displayed with protein sediment, asthenia, Cr 5.9 mg/dL, urea 121 mg/dL, albumin 36 g/L, and proteinuria >300 mg/dL. At the time of this study, he showed a profile of dyslipidemia with hyperuricemia at 8.8 mg/dL. Immunological tests (IgG, IgG, IgA, ANA, and ANCA) were normal. Serology tests (VHB/VHC/VIH) were negative. Echography revealed hyperechogenic kidneys with low cortico-medullar differentiation and cortical slimming, and one simple renal cyst. He was transplanted at age 36 and required peritoneal hemodialysis ten years later. The second renal transplant was performed at age 54. His father experienced chronic kidney disease stage 3a3bA2 at the age of 81, with a history of hypertension, dyslipidemia, diabetes mellitus type 2, and obesity grade 1. His mother passed away at the age of 60 due to breast cancer. He has two siblings, a 54-year-old sister and a 46-year-old brother, who have normal FGe. It is worth noting that both the index patient and his father are being followed-up with because of familiar tremor with rigid-kinetic profile. He has two children, aged 16 and 13, who are apparently healthy.
– Case 4: A 39-year-old female patient with recurrent tonsillitis and urinary infections who presented with nephrotic syndrome at the age of 15. At the moment of diagnosis, proteinuria level was 96 mg/m^2^/h, accompanied by hypoalbuminemia, hyperlipidemia, microhematuria, and normal renal function with a creatinine level of 0.56 mg/dL. Immunological tests (IgG, IgM, IgA, ANA, C2, and C4) were normal. Renal echography revealed no alterations in size and differentiation. Renal biopsy was compatible with FSGS, showing global hyalinosis in 15% of glomeruli. She was started on treatment with glucocorticoids, cyclosporin, and ACE inhibitors with partial response reducing proteinuria up to 0.5 g/24 h. At age 18, immunosuppressors were removed, keeping normal renal function at Cr 0.7 mg/dL and proteinuria at 1.6 g/24 h. Her renal function worsened on follow-up at age 29, with Cr 1.2 mg/dL, proteinuria increasing to 3.5 g/24 h, and hypoalbuminemia. During the following year, the deterioration of renal function persisted, with Cr at 1.38 mg/dL and proteinuria at 5.7 g/24 h. A new renal biopsy revealed the progression of FSGS up to 40% of glomeruli, with renal atrophy, interstitial fibrosis associated with severe interstitial infiltrate, and glomerular deposits with segmentary distribution of IgM (3/4), C3, C4, C1q (2/4), and arteriolar deposits of C3. She did not respond to rituximab and plasmapheresis, leading to hemodialysis at age 31. Genetic testing of genes NPHS2, TRPC6, IFN2, and ACTN was negative. At age 32, she received a transplant from a live kidney donor and was treated with rituximab, plasmapheresis, thymoglobulin, and triple therapy with corticoids, tacrolimus, and mycophenolate due to thrombotic microangiopathy. Her father had onset kidney disease at 38 years old, with chronic kidney disease (creatinine 2.4 mg/dL GFR 35 mL/min) and proteinuria 2.4 g/24 h without hematuria. The immunological tests ANA and ANCA were negative and the complement C3 and C4 were normal. The kidney biopsy had sclerotic glomeruli, with mesangial matrix expansion with lobulation, in silver staining the walls of capillary loops and basement membranes were thickened, and doble contours were evident. Also, there was extensive tubular atrophy and nonspecific interstitial mononuclear infiltration. In the immunofluorescence labeling showing the presence of C3 and IgM pseudolinear deposits, the images are doubtful due to the advanced nature of the lesions described. After reviewing the renal biopsy, the pathologist cannot classify the glomerular disease, indicating lesions with double contours and increased lobularity, which suggests a pattern of membranoproliferative glomerulonephritis and glomerular sclerosis lesions. He subsequently began dialysis 3 years later, without receiving any type of immunosuppression. Her paternal grandmother deceased by renal disease at 32 years old, and her great-grandmother too died young.

### 3.2. Genetic Results and Clinical Interpretation

We identified four novel truncating mutations in the *PODXL* gene found in four Spanish index cases affected by glomerular disease, which were considered likely pathogenic. They represented 0.48% of primary causes of renal failure in the selected cohort. Further, 24% of the 818 patients had a positive genetic finding associated with a genetic disorder, which explained renal failure; thus, 2% of positive cases were caused by PODXL-podocytopathy.

All the patients affected by monoallelic variants in the *PODXL* gene had manifested end-stage kidney disease (ESKD) before the fourth decade of life, with the onset of urinary alterations (mainly proteinuria between 1–6 g/day) more than two decades earlier. Details on their clinical summaries and mutation types are described in Table 2. The four patients were heterozygous for a potentially disease-causing variant in the *PODXL* gene: p.Arg190* (case 1), p.Ile380Serfs*12 (case 2), p.Leu479Serfs*18 (case 3), and p.Gln494Alafs*16 (case 4), classified as likely pathogenic according to ACMG criteria [16]. The common criteria applied to classify the four variants were as follows: PVS1_very strong (8 points) and PM2_moderate (2 point). PVS1 is considered null variants (nonsense, frameshift, canonical ±1 or 2 splice sites, initiation codon, and single or multiexon deletion) in a gene where LOF is a known disease mechanism. PM2 is used when the variant is absent from controls (or is present at an extremely low frequency if recessive) in the Genomes Project Aggregation Consortium [4]. All the variants were truncating variants predicted to introduce a premature translation. Nonsense variant p.Arg190* was predicted to disrupt protein translation, leading to the loss of 66% of the normal protein mass (558 amino acids), including the whole cytoplasmic and transmembrane regions and part of the extracellular domain. The remaining three variants were frameshift and introduced a premature stop codon at the end of the extracellular domain (p.Ile380Serfs*12), the transmembrane, and the cytoplasmic domain (p.Leu479Serfs*18 and p.Gln494Alafs*16), which were predicted to escape the NMD mechanism. Therefore, the adaptive ACMG criteria by ClinGen recommendations proposed graduating PVS1 in strong (variant removes >10% of the protein) and moderate (variant removes <10% of the protein) [17]. Thus, the variants p.Ile380Serfs*12 and p.Leu479Serfs*18 reached PVS1_strong, whereas the variant p.Gln494Alafs*16 reached PVS1_moderate due to it being predicted to remove 9% of the protein. We extended the common criteria with the criterium PP1 about variant segregation in the families in which these data were available, applicating PP1_supporting. List of ACMG criteria are shown in Table 1.

**Figure 2:**
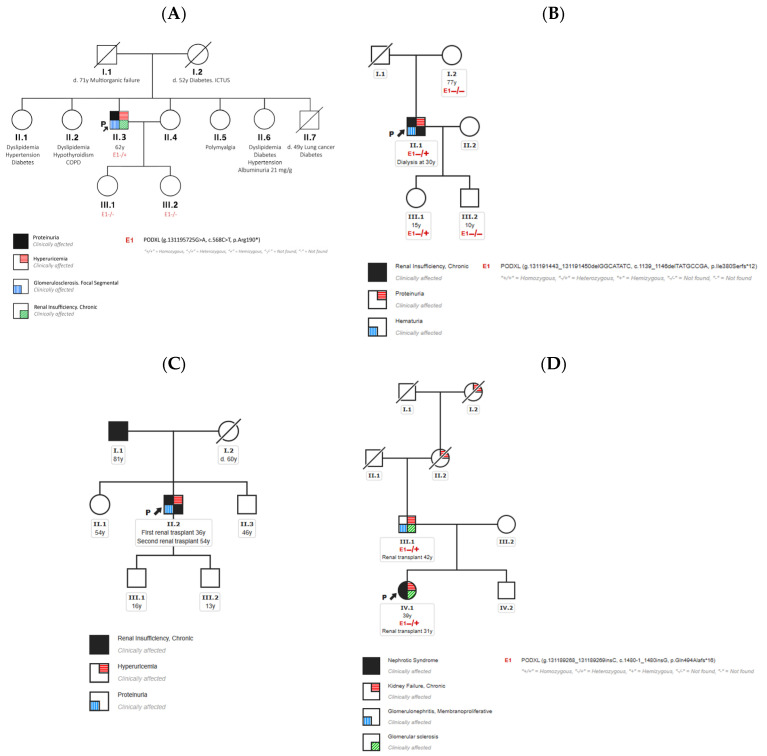
Family tests and pedigrees of the families reported in this study. Blank symbols = unaffected; E1−/− = wild-type variant; and E1−/+ = heterozygous variant. (**A**) Family tree of case 1; (**B**) family tree of case 2; (**C**) family tree of case 3; and (**D**) family tree of case 4.

Genetic testing was performed on the available family members with the purpose of studying the segregation of the selected variant in the *PODXL* gene with the disease in the four cases (Figure 2). The variant found in case 1 could be tested in his daughters, both of whom carried the wild type for the variant. The variant identified in case 2 was absent in his unaffected mother and in one unaffected 10-year-old son, while it was transmitted to his 15-year-old daughter, who was unaffected at the moment of this study. The variant detected in case 4 was present in the affected father and absent in his asymptomatic brother, consistently with the autosomal dominant inheritance pattern in the family. Genetic testing could not be performed in any family members of case 3.

### 3.3. Cases from the Literature

After our review of the literature and analysis of data from specific databases (ClinVar and LOVD), we compiled 18 patients for whom we could extract phenotypic features and genetic results about variants in the PODXL gene (Table 2). Five unrelated families were revised from the original publications, including three nonsense variants (p.Gln485*, p.Arg326*, and p.Ser378*), one frameshift (pLys161Glufs*14), one missense (p.Leu474Arg), and one splicing (c.1101+2T>C) variant. In addition to the splicing variant, which was found in homozygosity in the LOVD database, the remaining previously described mutations were found in heterozygous patients diagnosed with FSGS and with suspected glomerular disease with proteinuria and elevated albumin and/or creatinine levels.

Almost all the heterozygous patients described in the literature had a positive family history for chronic kidney disease (CKD) with the segregation of the variant among family members. Taking into account the whole set of patients, including the four reported in this study, we collected 22 individuals (11 females and 11 males) with an average of renal disease onset at age 22.1. 80% of the patients older than age 35 developed ESKD (Table 2). Although all the patients had suspected glomerular disease, renal biopsy was performed in 40% of the patients, according to each publication, and FSGS was observed when renal biopsy was available. Tubular atrophy and membranoproliferative glomerulonephritis were present in three individuals. We did not consider the patients with biallelic LoF variants, reported in the scientific literature and in LOVD, for their inclusion in the statistics of the whole cohort, due to the absence of clinical data.

### 3.4. Comparative Cohort

With the purpose of comparing the frequency of truncating variants in other cohorts of non-renal patients, we analyzed the frequency of truncating mutations in the PODXL gene (nonsense, frameshift, and splicing variants affecting canonical positions) in an internal cohort of 4408 individuals sequenced by high-throughput sequencing (Table 3). This cohort was divided into two groups: 1663 patients referred for genetic testing due to renal diseases and 2745 patients with non-renal diseases, including cardiovascular, neurological, and immunological disorders. Among the total number of patients from this cohort, four individuals carried potential truncating variants in the PODXL gene (one frameshift and three splicing variants).

Two variants were identified in adult patients who presented with CKD for which no other genetic cause was determined, while the remaining two variants were detected in a young patient with the age of 15 years and a fetus, who were affected by non-renal diseases. In these patients, whole exome analysis was addressed to genes associated with the phenotype of osteomyelitis in one case, and congenital cardiomyopathy in the other case. The two variants presented in the renal patients were located in canonical splicing sites (c.100+1G>A and c.1480-1G>C), one was identified in a 50-year-old male patient with glomerulosclerosis, proteinuria, and a maternal family history of dialysis, whereas the other was found in a 63-year-old male with proteinuria, hyperuricemia, and progressive CKD. We did not extend the description of genetic and phenotype results of these two patients in this study.

Regarding the GENSEN cohort (818 patients), 17% (34 patients) had a genetic cause of podocytopathy. Among these patients, 11.7% (4/34) had PODXL-podocytopathy. Compared with the internal cohort of renal and non-renal patients, the GENSEN cohort showed enriched PODXL-truncating mutations with statistically significant data (Table 3).

### 3.5. Limitations of the Genetic Test

Other family members of the four index cases could not be tested for the variant of interest in each case, either because they were deceased or because there were limitations to collecting samples from patients without a positive family history.

## 4. Discussion

To date, only a few families have been reported to harbor pathogenic variants in the *PODXL* gene. All pathogenic variants described prior to this study were truncating mutations that introduced a premature translation stop codon, which has been demonstrated to cause protein haploinsufficiency or to disrupt its function. At the molecular level, mRNA from patients’ peripheral blood mononuclear cells (PBMCs) harboring the previously known variants p.Arg326* and p.Ser378* showed a significant reduction by real-time PCR compared with wild-type controls. This reduction was also observed by Western blot analysis, with PODXL levels decreased by half. A functional analysis performed using pEGFP-PODXL plasmids definitely confirmed that the mutant sequence underwent the nonsense-mediated mRNA decay (NMD) pathway, leading to protein haploinsufficiency [6]. At the tissue level, immunofluorescence assays using anti-PODXL antibodies from the glomerular basement membranes of patients harboring variant p.Gln485* did not reveal any significant changes in the semiquantitative analysis, which may suggest that this variant, located in the terminal part of the PODXL protein, could escape the NMD mechanism, and, thus, the protein would be expressed [7]. In addition, the mice models with a complete deficiency of *PODXL* exhibited the disorganization of renal architecture and kidney injury that cause perinatal lethality. It was extrapolated with the biallelic LoF variant identified in humans in relation to congenital nephrotic syndrome, which was reported in one publication [7] and in the LOVD database [18]. The evidence about the effect of PODXL-haploinsufficiency was first described in Podxl^+/−^ mice, whose histological examination showed normal glomerular and tubulointerstitial structures, but a slight reduction in the filtration properties of the podocytes with normal kidney function compared with wild-type mice [19]. On detailed examination, glomeruli were found to have segmental lesions and collapsing features, and urinalysis revealed proteinuria, elevated serum creatinine, and reduced albumin [19]. This phenotype was consistent with the one observed in humans carrying truncating mutations in *PODXL* gene, revealing the spectrum of clinical manifestations.

In this study, we reported four novel truncating variants in the *PODXL* gene. According to the previous discussion, two of the variants (p.Arg190*and p.Ile380Serfs*12) were predicted to undergo NMD mechanism leading to protein haploinsufficiency. NMD plays a very important role not only in understanding the biological cause of variant pathogenicity in genetic disease, but also helping to address gene therapies by increasing the production of full-length PODXL protein [20]. The remaining two novel variants p.Leu479Serfs*18 and p.Gln494Alafs*16 were located in the final 10% of the aminoacidic sequence. Following the molecular evaluation suggested in relation to the previously described variant, p.Gln485*, this result questions the involvement of the NMD escape mechanism when the premature stop codon is located in the terminal part of podocalyxin. We consider that both novel variants, p.Leu479Serfs*18 and p.Gln494Alafs*16, would need to be functionally studied in order to establish whether they can escape the NMD pathway and disrupt the function of the PODXL protein. Only one heterozygous missense variant was reported in the scientific literature proposed to cause familiar FSGS [21]. In this study, the variant p.Leu442Arg (also called p.Leu474Arg) affecting the transmembrane region of the protein segregated in affected family members with the disease but its full effect on protein function remains unknown, opening the possibility that other pathogenic mechanisms different than the ones caused by truncating variants may occur.

Notably, the tolerance of the *PODXL* gene to LoF variants (pLI = 0.27; 46.1 expected SNVs vs. 19 observed SNVs) is not too elevated compared with other full-penetrance genes with autosomal dominant inheritance that are intolerant to LoF variants. This score seems to be underestimated because of the numerous LoF variants in the first 31 amino acids reported in gnomAD. This region encompasses exon 1 of *PODXL*, which has an extremely high rate of GC (close to 80%), predisposing to the accumulation of artifacts and to frequent insertions and deletions. In addition, another methionine codon (Met56) is located in the proximal region of exon 2. To support this suggestion, we observed 103 different insertions/deletions, some of them found in more than one individual, in the amino acids between residues Leu19 and Ser31, and found that in-frame and frameshift mutations located in the first exon of *PODXL* are tolerated and a skip to the next methionine residue is likely to occur. This fact has been observed in genes in which nonsense variants located near the *N*-terminal region (among the first 55 amino acids) of the protein can bypass the NMD pathway, reinitiating translation by using an alternative start codon, generally in Met55 [22,23].

Interestingly, we detected 0.09% of pLoF variants (4 SNVs) in *PODXL* in an internal cohort of sequenced patients with renal and non-renal diseases, whereas 0.48% were observed in the GENSEN cohort, composed of only CKD patients. The GENSEN cohort showed statistically significant enrichment of PODXL truncating variants, consistently with the genetic cause of podocytopathies in this set of patients. Two of the patients from the internal cohort were adult individuals who showed the expected phenotype for defects in the *PODXL* gene without any other genetic cause of the disease, whereas the remaining two patients harboring truncating mutations in *PODXL* were young individuals without a renal phenotype at the moment of this study. The evaluation of these other patients potentially affected by disease-causing *PODXL* mutations was not investigated in this report, but it is expected that adult patients with monoallelic truncating variants could be affected by the entity proposed in this study.

We carried out genetic testing on family members of our four cases. The results, although limited because of the low number of individuals included in family testing, revealed cosegregation in the affected families, following an autosomal dominant pattern in the family of case 4. We did not find any asymptomatic adult carriers of any of the studied variants in the *PODXL* gene; therefore, the presence of highly penetrant LoF mutations with a late onset is not ruled out, which could explain an incomplete PODXL pLI score. We did not carry out the follow-up of young patients who harbored monoallelic truncating variants, so we do not have enough data to evaluate the penetrance of variants in this gene. Genetic testing could not be performed in a large number of family members to strictly determine whether variants were inherited or de novo in all cases.

Clinically, patients harboring monoallelic truncating mutations in the *PODXL* gene displayed the onset of renal disease during their third decade of life and experienced alterations on urinalysis, such as proteinuria and/or elevated serum creatinine and albumin levels. ESKD was observed in 80% of patients older than age 35 (8/10), whereas it was observed in 41.7% of patients under age 35, suggesting a rapid progression of renal disease in adults from age 35 onwards. The reduced number of reported cases limits the accuracy of the data about the behavior of the disorder in the context of kidney failure; in fact, case 1 of this study did not progress to ESKD after 60 years of age. FSGS was the most frequent finding in biopsies when available, suggesting that genetic testing could replace biopsy in patients affected by this podocytopathy. Although podocalyxin regulates murine vascular permeability and its deficiency alters endothelial barrier with vascular lesions [10], this phenotype was not observed in patients with monoallelic variants in *PODXL*. We cannot discard affection in the pulmonary vasculature when *PODXL* is completely lost. We propose that *PODXL* haploinsufficiency represents a molecular entity of podocytopathies that is associated with an autosomal dominant FSGS with adult onset and variable progression of kidney deterioration. Patients with other familial podocytopathies, such as *ACTN4*, *CD2AP,* or *LMX1B,* showed variable histological phenotypes in the biopsies including membranous proliferative glomerulonephritis (MPGN), dense deposits, and tubular atrophy, suggesting a wide spectrum of glomerular alterations apart from FSGS [24,25]. In some individuals affected by the *PODXL* condition, biopsies revealed borderline forms of FSGS and MPGN [7]; thus, further histological studies would be necessary to provide more accurate data about the physiopathology associated with *PODXL* defects. In addition to LoF variants, the overexpression of podocalyxin has been positively correlated with lupus nephritis (LN) disease, suggesting that different molecular mechanisms may occur [26].

## 5. Conclusions

In conclusion, we provide new evidence regarding the relevance of monoallelic LoF variants in the *PODXL* gene, highlighting the importance of exploring a new genetic disorder of podocytopathy, which needs to be included in glomerular disease panels and in human genetic disorder databases. Further studies about affected families are necessary to improve the clinical understanding and prognosis of this disorder, which is currently underdiagnosed. The specific location of each *PODXL* mutation is also critical for interpreting its significance and relevance in order to predict haploinsufficiency or abnormal function of the PODXL protein. The new findings reported in this study offer relevant clinical data, opening further opportunities for therapy research.

## Figures and Tables

**Table 1:** List of variants reported in this study. The localization of cDNA and protein were referred to the human reference GRCh37/hg19. ACMG criteria were adjusted considering ClinGen recommendations.

Patient	Sex	Onset of CKD	Onset of ESRD	Age at Study	Diagnosis	cDNA (NM_001018111.2)	Protein Change (NP_001018121.1)	Location of Stop Codon	Pathogenicity (ACMG Criteria)
Case 1	Male	40	no	62	FSGS/TA	c.568C>T	p.Arg190*	Extracellular domain	Likely pathogenic (PVS1_very strong; PM2_moderate)
Case 2	Female	22	30	50	GD	c.1139_1146delTATGCCGA	p.Ile380Serfs*12	Extracellular domain	Likely pathogenic (PVS1_strong; PM2_moderate)
Case 3	Male	44	44	55	GD	c.1435delC	p.Leu479Serfs*18	Transmembrane domain	Likely pathogenic (PVS1_strong; PM2_moderate), PP1_supporting
Case 4	Female	18	31	38	FSGS	c.1480-1_1480insG	p.Gln494Alafs*16	Cytoplasmic domain	Likely pathogenic (PVS1_moderate; PM2_moderate), PP1_supporintg

**Table 2:** Clinical synopsis of the 22 patients included in this study who harbored monoallelic mutations in the *PODXL* gene.

Clinical Summary	
Number of patients with monoallelic PODXL mutations	22
No. of unrelated families	9
Mean age of renal disease onset (years)	22.1
Patients with ESKD (%)	57.1
Mean age of patients with ESKD (years)	33.2
No. of patients with ESKD ≤ 35 years (%)	5/12 (41.7%)
No. of patients with ESKD > 35 years (%)	8/10 (80%)
Families with positive renal history (%)	66.7

**Table 3:** Cohort comparison between loss-of-function (LoF) variants from the GENSEN cohort and our internal patient cohort. The GENSEN cohort included CKD patients, whereas our internal cohort included patients with renal and non-renal phenotypes.

Cohorts	LoF Variants in PODXL	Total Patients (%)	OR	*p*-Value (CI)
GENSEN cohort	4	818 (0.48)	5.38	0.024 (1–29)
Internal cohort of patients	4	4408 (0.09)		

## Data Availability

The original contributions presented in the study are included in the article/Supplementary Material, further inquiries can be directed to the corresponding author.

## Supplementary Materials

The following supporting information can be downloaded at https://www.mdpi.com/article/10.3390/genes16040464/s1: Table S1: List of patients harboring monoallelic variants in *PODXL* gene. Diagnostics: “FSGS” Focal Segmentary Glomerulosclerosis; “MPGN” Membrane Proliferative Glomerulonephritis; “GD” Glomerular Disease”; and Nephrotic syndrome “NS”.

## Abbreviations

The following abbreviations are used in this manuscript:

ESKD	End-stage kidney disease
CKD	Chronic kidney disease
HTS	High-throughput sequencing
FSGS	Focal segmental glomerulosclerosis
