# Supplementary material for: Novel Truncating Variants in PODXL Represent a New Entity to Be Explored Among Podocytopathies
Source: Genes (Basel). 2025 Apr 18;16(4):464. doi: 10.3390/genes16040464 (PMC12026838; doi:10.3390/genes16040464)
Supplement: Supplementary file 1 [file genes-16-00464-s001.zip › genes-3551580-supplementary.pdf]

Patients	Pedigree status	Sex	Onset of CKD	Age at study	Age at ESRD	Proteinuria g/day (age)	Albumin level (g/L)	Creatinine	Diagnosis	Variant position	Family history	Population (country)	Pathogenicity (ACMG criteria)	Source
**Case 1**	Index	Male	40	62	no	1-2 (40)	normal	normal	FSGS	c.568C>T:p.Arg190*	Negative	Spain	LP (PVS1_very strong; PM2_moderate)	This study
**Case 2**	Index	Female	22	50	30	2,7		Elevated	GD	c.1139_1146delTATGCCGA: p.Ile380Serfs*12	Negative	Spain	LP (PVS1_strong; PM2_moderate)	This study
**Case 3**	Index	Male	<45	55	<45				GD	c.1435delC:p.Leu479Serfs*18	N/A	Spain	LP (PVS1_strong; PM2_moderate), PP1_supporting	This study
**Case 4**	Index	Female	18	38	31	6 (38)	N/A	N/A	FSGS	c.1480-1_1480insG:p.Gln494Alafs*16	Positive	Spain	LP (PVS1_moderate; PM2_moderate), PP1_supporintg	This study
**Barua (2014)**	Index	Male	26	59	<55	Proteinuria	N/A	Elevated	FSGS	c.1421T>G; p.Leu474Arg (L442R)	Positive	USA	VUS hot (PM2_supporting; PP1_moderate; PP3_moderate)	PMID:24048372
**Barua (2014)**	Sibling	Male	19	54	23	Proteinuria	N/A	Elevated	FSGS	c.1421T>G; p.Leu474Arg (L442R)	Positive	USA	VUS hot (PM2_supporting; PP1_moderate; PP3_moderate)	PMID:24048372
**Barua (2014)**	Daugther	Female	12	24	13	4 (12)	N/A	Elevated	GD	c.1421T>G; p.Leu474Arg (L442R)	Positive	USA	VUS hot (PM2_supporting; PP1_moderate; PP3_moderate)	PMID:24048372
**Barua (2014)**	Daugther	Female	N/A	26	No	4			GD	c.1421T>G; p.Leu474Arg (L442R)	Positive	USA	VUS hot (PM2_supporting; PP1_moderate; PP3_moderate)	PMID:24048372
**Barua (2014)**	Nephew	Female	13	26	No	Proteinuria			GD	c.1421T>G; p.Leu474Arg (L442R)	Positive	USA	VUS hot (PM2_supporting; PP1_moderate; PP3_moderate)	PMID:24048372
**Barua (2014)**	Nephew	Female	N/A	24	No				Unaffected	c.1421T>G; p.Leu474Arg (L442R)	Positive	USA	VUS hot (PM2_supporting; PP1_moderate; PP3_moderate)	PMID:24048372
**Barua (2014)**	Nephew	Female	N/A	20	No	3			GD	c.1421T>G; p.Leu474Arg (L442R)	Positive	USA	VUS hot (PM2_supporting; PP1_moderate; PP3_moderate)	PMID:24048372
**Lin (2019)**	Index	Male	30	72	39	Proteinuria	Elevated	Elevated	GD	c.976C>T; p.Arg326*	Positive	China	P (PVS1_very strong; PM2_moderate; PP1_supporting)	PMID:30523047
**Lin (2019)**	Sibling	Male	22	69	55	Proteinuria	Elevated	Elevated	GD	c.976C>T; p.Arg326*	Positive	China	P (PVS1_very strong; PM2_moderate; PP1_supporting)	PMID:30523047
**Lin (2019)**	Son	Male	25	39	72	Proteinuria	Elevated	Elevated	GD	c.976C>T; p.Arg326*	Positive	China	P (PVS1_very strong; PM2_supporting; PP1_supporting)	PMID:30523047
**Lin (2019)**	Son	Male	33	35	no	Proteinuria	Elevated	Elevated	GD	c.976C>T; p.Arg326*	Positive	China	P (PVS1_very strong; PM2_moderate; PP1_supporting)	PMID:30523047

**Lin (2019)**	Index	Male	56	62	no	Proteinuria	Elevated	Elevated	FSGS	c.1133C>G; p.Ser378*	Positive	India	P (PVS1_very strong; PM2_moderate; PP1_supporting)	PMID:30523047
**Lin (2019)**	Son	Male	20	26	no	Proteinuria	Elevated	Elevated	FSGS	c.1133C>G; p.Ser378*	Positive	India	P (PVS1_very strong; PM2_moderate; PP1_supporting)	PMID:30523047
**Lin (2019)**	Daugther	Female	26	31	no	Proteinuria	Elevated	Elevated	FSGS	c.1133C>G; p.Ser378*	Positive	India	P (PVS1_very strong; PM2_moderate; PP1_supporting)	PMID:30523047
**Marx (2021)**	Index	Female	10	35	19	>3 (10)			NS	c.1453C>T; p.Gln485*	Positive	France	LP (PVS1_moderate; PM2_moderate), PP1_suppo329intg	PMID:33780168
**Marx (2021)**	Daugther	Female	12	25	25	1.5 (25)	38		FSGS vs MPGN	c.1453C>T; p.Gln485*	Positive	France	LP (PVS1_moderate; PM2_moderate), PP1_supporintg	PMID:33780168
**Marx (2021)**	Daugther	Female	12	25	25	4 (25)	37		FSGS vs MPGN	c.1453C>T; p.Gln485*	Positive	France	LP (PVS1_moderate; PM2_moderate), PP1_supporintg	PMID:33780168
**Zeni (2023)**	Index	Male	1	3	-	Proteinuria	Hypoalbuminemia	Elevated	NS	c.480dupG; p.Lys161Glufs*14	N/A	Italy	LP (PVS1_very strong; PM2_supporting)	PMID: 38156539

Table 1 (supplementary material): List of patients harboring monoallelic variants in *PODXL* gene. Diagnostics: “FSGS” Focal Segmentary Glomerulosclerosis; “MPGN” Membrane Proliferative Glomerulonephritis; “GD” Glomerular Disease”; Nephrotic syndrome “NS”.
